# Supplementary material for: A systematic review of alternative surveillance approaches for lymphatic filariasis in low prevalence settings: Implications for post-validation settings
Source: PLoS Negl Trop Dis. 2020 May 12;14(5):e0008289. doi: 10.1371/journal.pntd.0008289 (PMC7217451; doi:10.1371/journal.pntd.0008289)
Supplement: S2 File — (DOCX) [file pntd.0008289.s002.docx]

**S2 File. Search strategy**

| 1 | Elephantiasis, filarial [MeSH term] |
| --- | --- |
| 2 | Elephantiasis [All fields] |
| 3 | Filarial [All fields] |
| 4 | 2 AND 3 |
| 5 | Filarial elephantiasis [All fields] |
| 6 | Lymphatic filariasis [All fields] |
| 7 | Filariasis [All fields] |
| 8 | Lymphatic [All fields] |
| 9 | 7 OR 8 |
| 10 | 1 OR 4 OR 5 OR 6 OR 9 |
| 11 | Surveillance [All fields] |
| 12 | Survey [All fields] |
| 13 | Monitoring [All fields] |
| 14 | Xenomonitoring [All fields] |
| 15 | 11 OR 12 OR 13 OR 14 |
| 16 | 9 AND 16 |
| 17 | Remove duplicates from 16 |
