# Supplementary material for: A systematic review of alternative surveillance approaches for lymphatic filariasis in low prevalence settings: Implications for post-validation settings
Source: PLoS Negl Trop Dis. 2020 May 12;14(5):e0008289. doi: 10.1371/journal.pntd.0008289 (PMC7217451; doi:10.1371/journal.pntd.0008289)
Supplement: S1 Table — (DOCX) [file pntd.0008289.s003.docx]

| **S1 Table. Risk of bias assessment for human and mosquito studies** | | | | | | | | | | |
| --- | --- | --- | --- | --- | --- | --- | --- | --- | --- | --- |
| **A: Human studies** | | | | | | | | | | |
| **AUTHOR** | **COUNTRY** | **SAMPLE SIZE** | | **PARTICIPANT SAMPLING** | | **STUDY DESIGN** | | **STUDY POPULATION** | | **TOTAL SCORE** |
|  |  | **N** | **Score** | **Type** | **Score** | **Type** | **Score** | **Children AND adults?** | **Score** |  |
| Coutts et al. (2017) [1] | American Samoa | 1,881 | 1 | Random | 2 | Cross-sectional | 1 | Yes | 2 | **6** |
| Lau et al. (2014) [2] | American Samoa | 807 | 1 | Random | 2 | Cross-sectional | 1 | No | 1 | **5** |
| Lau et al. (2017a) [3] | American Samoa | 602 | 0 | Non-random | 1 | Cross-sectional | 1 | Yes | 2 | **4** |
| Lau et al. (2017b) [3] | American Samoa | 476 | 0 | Non-random | 1 | Cross-sectional | 1 | Yes | 2 | **4** |
| Lau et al. (2017c) [3] | American Samoa | 283 | 0 | Non-random | 1 | Cross-sectional | 1 | No | 1 | **3** |
| Mladonicky et al. (2009) [4] | American Samoa | 579 | 0 | Non-random | 1 | Cross-sectional | 1 | Yes | 2 | **4** |
| Sheel et al. (2018) [5] | American Samoa | 2,507 | 2 | Random | 2 | Cross-sectional | 1 | Yes | 2 | **7** |
| Won et al. (2018) [6] | American Samoa | 1,998 | 1 | Non-random | 1 | Longitudinal | 2 | No | 1 | **5** |
| Huang et al. (2016a) [7] | China | 542 | 0 | Not stated | 0 | Cross-sectional | 1 | No | 1 | **2** |
| Huang et al. (2016b) [7] | China | 436 | 0 | Not stated | 0 | Cross-sectional | 1 | NS | 0 | **1** |
| Huang et al. (2016c) [7] | China | 5,787 | 2 | Not stated | 0 | Cross-sectional | 1 | NS | 0 | **3** |
| Huang et al. (2016d) [7] | China | 762 | 1 | Not stated | 0 | Cross-sectional | 1 | Yes | 2 | **4** |
| Huang et al. (2016e) [7] | China | 218 | 0 | Not stated | 0 | Longitudinal | 2 | NS | 0 | **2** |
| Itoh et al. (2007) [8] | China | 10,409 | 2 | Not stated | 0 | Cross-sectional | 1 | No | 1 | 4 |
| Moustafa et al. (2014a) [9] | Egypt | 1,321 | 1 | Non-random | 1 | Cross-sectional | 1 | No | 1 | 4 |
| Moustafa et al. (2014b) [9] | Egypt | 75 | 0 | Non-random | 1 | Cross-sectional | 1 | No | 1 | 3 |
| Ramzy et al. (2006a) [10] | Egypt | 1,808 | 1 | Random | 2 | Longitudinal | 2 | Yes | 2 | 7 |
| Ramzy et al. (2006b) [10] | Egypt | 1,653 | 1 | Not stated | 0 | Longitudinal | 2 | No | 1 | 4 |
| Gass et al. (2011a) [11] | French Polynesia | 1,383 | 1 | Non-random | 1 | Cross-sectional | 1 | Yes | 2 | 5 |
| Won et al. (2018) [12] | Gambia | 2,612 | 2 | Non-random | 1 | Cross-sectional | 1 | Yes | 2 | 6 |
| Gass et al. (2011b) [11] | Ghana | 1,466 | 1 | Non-random | 1 | Cross-sectional | 1 | Yes | 2 | 5 |
| Owusu et al. (2015a) [13] | Ghana | 308 | 0 | Random | 2 | Cross-sectional | 1 | No | 1 | 4 |
| Owusu et al. (2015b) [13] | Ghana | 653 | 0 | Random | 2 | Cross-sectional | 1 | Yes | 2 | 5 |
| Gass et al. (2011c) [11] | Haiti | 1,322 | 1 | Non-random | 1 | Cross-sectional | 1 | Yes | 2 | 5 |
| Mehta et al. (2018) [14] | India | 290 | 0 | Not stated | 0 | Cross-sectional | 1 | Yes | 2 | 3 |
| Ramaiah et al. (2013) [15] | India | 700 | 0 | Random | 2 | Longitudinal | 2 | Yes | 2 | 6 |
| Swaminathan et al. (2017) [16] | India | 35,582 | 2 | Non-random | 1 | Cross-sectional | 1 | Yes | 2 | 6 |
| Garchitorena et al. (2018) [17] | Madagascar | 545 | 0 | Random | 2 | Cross-sectional | 1 | Yes | 2 | 5 |
| Coulibaly et al. (2015) [18] | Mali | 760 | 0 | Non-random | 1 | Longitudinal | 2 | Yes | 2 | 5 |
| Coulibaly et al. (2016a) [19] | Mali | 3,457 | 2 | Non-random | 1 | Longitudinal | 2 | No | 1 | 6 |
| Coulibaly et al. (2016b)[19] | Mali | 1,184 | 1 | Not stated | 0 | Longitudinal | 2 | Yes | 2 | 5 |
| Richards et al. (2011) [20] | Nigeria | 1,720 | 1 | Non-random | 1 | Longitudinal | 2 | Yes | 2 | 6 |
| Mitja et al. (2011) [21] | Papua New Guinea | 6,263 | 2 | Not stated | 0 | Longitudinal | 2 | Yes | 2 | 6 |
| Joseph et al. (2011A) [22] | Samoa | 6,648 | 2 | Random | 2 | Cross-sectional | 1 | Yes | 2 | 7 |
| Joseph et al. (2011Ba) [23] | Samoa | 2,474 | 2 | Random | 2 | Cross-sectional | 1 | Yes | 2 | 7 |
| Harrington et al. (2013) [24] | Solomon Islands | 307 | 0 | Non-random | 1 | Cross-sectional | 1 | Yes | 2 | 4 |
| Chandrasena et al. (2016a) [25] | Sri Lanka | 2,461 | 1 | Non-random | 1 | Longitudinal | 2 | Yes | 2 | 6 |
| Chandrasena et al. (2016b) [25] | Sri Lanka | 250 | 0 | Not stated | 0 | Cross-sectional | 1 | No | 1 | 2 |
| Gass et al. (2011d) [11] | Sri Lanka | 1,477 | 1 | Non-random | 1 | Cross-sectional | 1 | Yes | 2 | 5 |
| Rahman et al. (2018a) [26] | Sri Lanka | 630 | 0 | Non-random | 1 | Cross-sectional | 1 | Yes | 2 | 4 |
| Rahman et al. (2018b) [26] | Sri Lanka | 2,301 | 1 | Non-random | 1 | Cross-sectional | 1 | No | 1 | 4 |
| Rao et al. (2014a) [27] | Sri Lanka | 7,156 | 2 | Random | 2 | Cross-sectional | 1 | Yes | 2 | 7 |
| Rao et al. (2014b) [27] | Sri Lanka | 17,000 | 2 | Non-random | 1 | Cross-sectional | 1 | No | 1 | 5 |
| Rao et al. (2016) [28] | Sri Lanka | 12,977 | 2 | Random | 2 | Cross-sectional | 1 | Yes | 2 | 7 |
| Rao et al. (2017a) [29] | Sri Lanka | 2,227 | 1 | Non-random | 1 | Cross-sectional | 1 | No | 1 | 4 |
| Rao et al. (2017b) [29] | Sri Lanka | 3,123 | 2 | Random | 2 | Cross-sectional | 1 | Yes | 2 | 7 |
| Rao et al. (2018a) [30] | Sri Lanka | 401 | 0 | Non-random | 1 | Cross-sectional | 1 | No | 1 | 3 |
| Rao et al. (2018b) [30] | Sri Lanka | 528 | 0 | Random | 2 | Cross-sectional | 1 | Yes | 2 | 5 |
| Rao et al. (2018c) [30] | Sri Lanka | 16,927 | 2 | Random | 2 | Cross-sectional | 1 | Yes | 2 | 7 |
| Gass et al. (2011e) [11] | Tanzania | 1,384 | 1 | Non-random | 1 | Cross-sectional | 1 | Yes | 2 | 5 |
| Jones et al. (2018) [31] | Tanzania | 854 | 1 | Random | 2 | Cross-sectional | 1 | Yes | 2 | 6 |
| Budge et al. (2014a) [32] | Togo | 6,509 | 2 | Non-random | 1 | Longitudinal | 2 | No | 1 | 6 |
| Budge et al. (2014b) [32] | Togo | 7,800 | 2 | Non-random | 1 | Cross-sectional | 1 | No | 1 | 5 |
| Budge et al. (2014c) [32] | Togo | 2,880 | 2 | Non-random | 1 | Longitudinal | 2 | No | 1 | 6 |
| Dorkenoo et al. (2018) [33] | Togo | 40 | 0 | Non-random | 1 | Cross-sectional | 1 | Yes | 2 | 4 |
| Mathieu et al. (2011) [34] | Togo | 8,050 | 2 | Non-random | 1 | Longitudinal | 2 | NS | 0 | 5 |
| Joseph et al. (2011Bb) [23] | Tonga | 797 | 1 | Non-random | 1 | Cross-sectional | 1 | No | 1 | 4 |
| Gass et al. (2011f) [11] | Tuvalu | 1,481 | 1 | Non-random | 1 | Cross-sectional | 1 | Yes | 2 | 5 |
| Allen at al. (2017) [35] | Vanuatu | 7,657 | 2 | Random | 2 | Cross-sectional | 1 | Yes | 2 | 7 |
| Joseph et al. (2011Bc) [23] | Vanuatu | 3,840 | 2 | Non-random | 1 | Cross-sectional | 1 | No | 1 | 5 |

| **B: Mosquito studies** | | | | | | | | | | |
| --- | --- | --- | --- | --- | --- | --- | --- | --- | --- | --- |
| **AUTHOR** | **COUNTRY** | **SAMPLE SIZE** | | **CATCHSITE SAMPLING** | | **STUDY DESIGN** | | **METHOD OF ANALYSIS** | | **TOTAL SCORE** |
|  |  | **N** | **Score** | **Method** | **Score** | **Type** | **Score** | **Type** | **Score** |  |
| Schmaedick (2014) [36] | American Samoa | 21,861 | 2 | Purposive | 1 | Cross-sectional | 1 | PCR analysis | 2 | 6 |
| Irish et al. (2018) [37] | Bangladesh | 5,926 | 1 | Random | 2 | Cross-sectional | 1 | PCR analysis | 2 | 6 |
| Abdel-Shafi et al. (2016) [38] | Egypt | Not stated | 0 | Random | 2 | Cross-sectional | 1 | PCR analysis | 2 | 5 |
| Moustafa et al. (2017) [39] | Egypt | 7,970 | 1 | Not stated | 0 | Cross-sectional | 1 | PCR analysis | 2 | 4 |
| Ramzy et al. (2006) [10] | Egypt | 8,531 | 1 | Random | 2 | Longitudinal | 2 | PCR analysis | 2 | 7 |
| Owusu et al. (2015a) [13] | Ghana | 401 | 0 | Random | 2 | Cross-sectional | 1 | PCR analysis | 2 | 5 |
| Owusu et al. (2015b) [13] | Ghana | 4,099 | 0 | Random | 2 | Cross-sectional | 1 | PCR analysis | 2 | 5 |
| Mehta et al. (2018) [14] | India | 2,429 | 0 | Purposive | 1 | Cross-sectional | 1 | Dissection | 1 | 3 |
| Ramaiah et al. (2013) [15] | India | 10,842 | 1 | Not stated | 0 | Longitudinal | 2 | Dissection | 1 | 4 |
| Subramanaian et al. (2017) [40] | India | 41,294 | 2 | Random | 2 | Longitudinal | 2 | PCR analysis | 2 | 8 |
| Ben et al. (2016) [41] | Malaysia | 4,378 | 0 | Not stated | 0 | Cross-sectional | 1 | PCR analysis | 2 | 3 |
| Coulibaly et al. (2015) [18] | Mali | 4,680 | 1 | Not stated | 0 | Longitudinal | 2 | Dissection | 1 | 4 |
| Coulibaly et al. (2016) [19] | Mali | 14,424 | 2 | Not stated | 0 | Longitudinal | 2 | Dissection | 1 | 5 |
| Coulibaly et al. (2016) [19] | Mali | 115 | 0 | Random | 2 | Longitudinal | 2 | PCR analysis | 2 | 6 |
| Richards et al. (2011) [20] | Nigeria | 4,398 | 0 | Convenience | 1 | Longitudinal | 2 | Dissection | 1 | 4 |
| Reimer et al. (2013) [42] | Papua New Guinea | 20,345 | 2 | Not stated | 0 | Cross-sectional | 1 | PCR analysis | 2 | 5 |
| Cho et al. (2012) [43] | South Korea | 5,380 | 1 | Not stated | 0 | Cross-sectional | 1 | PCR analysis | 2 | 4 |
| Rao et al. (2018) [30] | Sri Lanka | 7,750 | 1 | Random | 2 | Cross-sectional | 1 | PCR analysis | 2 | 6 |
| Rao et al. (2014) [27] | Sri Lanka | 69,680 | 2 | Random | 2 | Cross-sectional | 1 | PCR analysis | 2 | 7 |
| Rao et al. (2016) [28] | Sri Lanka | 28,717 | 2 | Random | 2 | Cross-sectional | 1 | PCR analysis | 2 | 7 |
| Rao et al. (2017c) [29] | Sri Lanka | 48,301 | 2 | Random | 2 | Longitudinal | 2 | PCR analysis | 2 | 8 |
| Jones et al. (2018) [31] | Tanzania | 1,650 | 0 | Purposive | 1 | Cross-sectional | 1 | PCR analysis and dissection | 2 | 4 |
| Dorkenoo et al. (2018) [44] | Togo | 10,872 | 2 | Random | 2 | Cross-sectional | 1 | PCR analysis | 2 | 7 |

**References**

1. Coutts SP, King JD, Pa'au M, Fuimaono S, Roth J, King MR, et al. Prevalence and risk factors associated with lymphatic filariasis in American Samoa after mass drug administration. Tropical Medicine and Health. 2017;45:22. Epub 2017/08/11. doi: 10.1186/s41182-017-0063-8. PubMed PMID: 28794687; PubMed Central PMCID: PMCPMC5543440.

2. Lau CL, Won KY, Becker L, Soares Magalhaes RJ, Fuimaono S, Melrose W, et al. Seroprevalence and spatial epidemiology of lymphatic filariasis in American Samoa after successful mass drug administration. PLoS Negl Trop Dis. 2014;8(11):e3297-e. doi: 10.1371/journal.pntd.0003297. PubMed PMID: 25393716.

3. Lau CL, Sheridan S, Ryan S, Roineau M, Andreosso A, Fuimaono S, et al. Detecting and confirming residual hotspots of lymphatic filariasis transmission in American Samoa 8 years after stopping mass drug administration. PLoS Negl Trop Dis. 2017;11(9):e0005914. doi: 10.1371/journal.pntd.0005914.

4. Mladonicky JM, King JD, Liang JL, Chambers E, Pa'au M, Schmaedick MA, et al. Assessing transmission of lymphatic filariasis using parasitologic, serologic, and entomologic tools after mass drug administration in American Samoa. Am J Trop Med Hyg. 2009;80(5):769-73. Epub 2009/05/02. PubMed PMID: 19407122.

5. Sheel M, Sheridan S, Gass K, Won K, Fuimaono S, Kirk M, et al. Identifying residual transmission of lymphatic filariasis after mass drug administration: Comparing school-based versus community-based surveillance - American Samoa, 2016. PLoS Negl Trop Dis. 2018;12(7):e0006583. doi: 10.1371/journal.pntd.0006583.

6. Won KY, Robinson K, Hamlin KL, Tufa J, Seespesara M, Wiegand RE, et al. Comparison of antigen and antibody responses in repeat lymphatic filariasis transmission assessment surveys in American Samoa. PLoS Negl Trop Dis. 2018;12(3):e0006347. doi: 10.1371/journal.pntd.0006347.

7. Huang BC, Li J, Hu YX, Duan JH, Yin K, Xiao T, et al. Study on application of filarial specific IgG4 kit in disease surveillance of lymphatic filariasis. Int J Clin Exp Med. 2016;9:4332-9.

8. Itoh M, Wu W, Sun D, Yao L, Li Z, Islam MZ, et al. Confirmation of elimination of lymphatic filariasis by an IgG4 enzyme-linked immunosorbent assay with urine samples in Yongjia, Zhejiang Province and Gaoan, Jiangxi Province, People's Republic of China. Am J Trop Med Hyg. 2007;77(2):330-3. Epub 2007/08/11. PubMed PMID: 17690407.

9. Moustafa MA, Thabet HS, Saad GA, El-Setouhy M, Mehrez M, Hamdy DM. Surveillance of lymphatic filariasis 5 years after stopping mass drug administration in Menoufiya Governorate, Egypt. East Mediterr Health J. 2014;20(5):295-9. Epub 2014/06/22. PubMed PMID: 24952286.

10. Ramzy RM, El Setouhy M, Helmy H, Ahmed ES, Abd Elaziz KM, Farid HA, et al. Effect of yearly mass drug administration with diethylcarbamazine and albendazole on bancroftian filariasis in Egypt: a comprehensive assessment. The Lancet. 2006;367(9515):992-9. Epub 2006/03/28. doi: 10.1016/s0140-6736(06)68426-2. PubMed PMID: 16564361.

11. Gass K, Beau de Rochars MVE, Boakye D, Bradley M, Fischer PU, Gyapong J, et al. A Multicenter Evaluation of Diagnostic Tools to Define Endpoints for Programs to Eliminate Bancroftian Filariasis. PLOS Neglected Tropical Diseases. 2012;6(1):e1479. doi: 10.1371/journal.pntd.0001479.

12. Won KY, Sambou S, Barry A, Robinson K, Jaye M, Sanneh B, et al. Use of Antibody Tools to Provide Serologic Evidence of Elimination of Lymphatic Filariasis in The Gambia. The American journal of tropical medicine and hygiene. 2018;98(1):15-20. Epub 01/01. doi: 10.4269/ajtmh.17-0371. PubMed PMID: 29165213.

13. Owusu IO, de Souza DK, Anto F, Wilson MD, Boakye DA, Bockarie MJ, et al. Evaluation of human and mosquito based diagnostic tools for defining endpoints for elimination of Anopheles transmitted lymphatic filariasis in Ghana. Trans R Soc Trop Med Hyg. 2015;109(10):628-35. Epub 2015/09/20. doi: 10.1093/trstmh/trv070. PubMed PMID: 26385935.

14. Mehta PK, Rauniyar R, Gupta BP. Microfilaria persistent foci during post MDA and the risk assessment of resurgence in India. Tropical Medicine and Health. 2018;46:25-. doi: 10.1186/s41182-018-0107-8. PubMed PMID: 30026669.

15. Ramaiah KD, Vanamail P. Surveillance of lymphatic filariasis after stopping ten years of mass drug administration in rural communities in south India. Trans R Soc Trop Med Hyg. 2013;107(5):293-300. Epub 2013/02/28. doi: 10.1093/trstmh/trt011. PubMed PMID: 23442572.

16. Swaminathan S, Perumal V, Adinarayanan S, Kaliannagounder K, Rengachari R, Purushothaman J. Epidemiological assessment of eight rounds of mass drug administration for lymphatic filariasis in India: Implications for monitoring and evaluation. PLoS Negl Trop Dis. 2012;6(11):e1926. doi: 10.1371/journal.pntd.0001926.

17. Garchitorena A, Raza-Fanomezanjanahary EM, Mioramalala SA, Chesnais CB, Ratsimbasoa CA, Ramarosata H, et al. Towards elimination of lymphatic filariasis in southeastern Madagascar: Successes and challenges for interrupting transmission. PLoS Negl Trop Dis. 2018;12(9):e0006780. doi: 10.1371/journal.pntd.0006780.

18. Coulibaly YI, Dembele B, Diallo AA, Konate S, Dolo H, Coulibaly SY, et al. The Impact of Six Annual Rounds of Mass Drug Administration on Wuchereria bancrofti Infections in Humans and in Mosquitoes in Mali. Am J Trop Med Hyg. 2015;93(2):356-60. Epub 2015/06/03. doi: 10.4269/ajtmh.14-0516. PubMed PMID: 26033027; PubMed Central PMCID: PMCPMC4530761.

19. Coulibaly YI, Coulibaly SY, Dolo H, Konate S, Diallo AA, Doumbia SS, et al. Dynamics of antigenemia and transmission intensity of Wuchereria bancrofti following cessation of mass drug administration in a formerly highly endemic region of Mali. Parasites & Vectors. 2016;9(1):628-. doi: 10.1186/s13071-016-1911-9. PubMed PMID: 27912789.

20. Richards FO, Eigege A, Miri ES, Kal A, Umaru J, Pam D, et al. Epidemiological and entomological evaluations after six years or more of mass drug administration for lymphatic filariasis elimination in Nigeria. PLoS Negl Trop Dis. 2011;5(10):e1346. Epub 2011/10/25. doi: 10.1371/journal.pntd.0001346. PubMed PMID: 22022627; PubMed Central PMCID: PMCPMC3191131.

21. Mitjà O, Paru R, Hays R, Griffin L, Laban N, Samson M, et al. The Impact of a Filariasis Control Program on Lihir Island, Papua New Guinea. PLOS Neglected Tropical Diseases. 2011;5(8):e1286. doi: 10.1371/journal.pntd.0001286.

22. Joseph H, Maiava F, Naseri T, Silva U, Lammie P, Melrose W. Epidemiological assessment of continuing transmission of lymphatic filariasis in Samoa. Ann Trop Med Parasitol. 2011;105(8):567-78. Epub 2012/02/14. doi: 10.1179/2047773211y.0000000008. PubMed PMID: 22325816; PubMed Central PMCID: PMCPMC4089807.

23. Joseph H, Maiava F, Naseri T, Taleo F, ake M, Capuano C, et al. Application of the Filariasis CELISA Antifilarial IgG(4) Antibody Assay in surveillance in lymphatic filariasis elimination programmes in the South Pacific. J Trop Med. 2011;2011:492023. Epub 2011/10/01. doi: 10.1155/2011/492023. PubMed PMID: 21961018; PubMed Central PMCID: PMCPMC3180782.

24. Harrington H, Asugeni J, Jimuru C, Gwalaa J, Ribeyro E, Bradbury R, et al. A practical strategy for responding to a case of lymphatic filariasis post-elimination in Pacific Islands. Parasites & Vectors. 2013;6(1):218. doi: 10.1186/1756-3305-6-218.

25. Chandrasena NT, Premaratna R, Samarasekera DS, de Silva NR. Surveillance for transmission of lymphatic filariasis in Colombo and Gampaha districts of Sri Lanka following mass drug administration. Trans R Soc Trop Med Hyg. 2016;110(10):620-2. Epub 2016/11/07. doi: 10.1093/trstmh/trw067. PubMed PMID: 27816936.

26. Rahman MA, Yahathugoda TC, Tojo B, Premaratne P, Nagaoka F, Takagi H, et al. A surveillance system for lymphatic filariasis after its elimination in Sri Lanka. Parasitol Int. 2019;68(1):73-8. Epub 2018/10/12. doi: 10.1016/j.parint.2018.10.003. PubMed PMID: 30308253.

27. Rao RU, Nagodavithana KC, Samarasekera SD, Wijegunawardana AD, Premakumara WD, Perera SN, et al. A comprehensive assessment of lymphatic filariasis in Sri Lanka six years after cessation of mass drug administration. PLoS Negl Trop Dis. 2014;8(11):e3281. Epub 2014/11/14. doi: 10.1371/journal.pntd.0003281. PubMed PMID: 25393404; PubMed Central PMCID: PMCPMC4230885.

28. Rao RU, Samarasekera SD, Nagodavithana KC, Punchihewa MW, Dassanayaka TD, P KDG, et al. Programmatic use of molecular xenomonitoring at the level of evaluation units to assess persistence of lymphatic filariasis in Sri Lanka. PLoS Negl Trop Dis. 2016;10(5):e0004722. Epub 2016/05/20. doi: 10.1371/journal.pntd.0004722. PubMed PMID: 27196431; PubMed Central PMCID: PMCPMC4873130.

29. Rao RU, Samarasekera SD, Nagodavithana KC, Dassanayaka TDM, Punchihewa MW, Ranasinghe USB, et al. Reassessment of areas with persistent lymphatic filariasis nine years after cessation of mass drug administration in Sri Lanka. PLoS Negl Trop Dis. 2017;11(10):e0006066. Epub 2017/10/31. doi: 10.1371/journal.pntd.0006066. PubMed PMID: 29084213; PubMed Central PMCID: PMCPMC5679644.

30. Rao RU, Samarasekera SD, Nagodavithana KC, Goss CW, Punchihewa MW, Dassanayaka TDM, et al. Comprehensive Assessment of a Hotspot with Persistent Bancroftian Filariasis in Coastal Sri Lanka. Am J Trop Med Hyg. 2018;99(3):735-42. Epub 2018/07/18. doi: 10.4269/ajtmh.18-0169. PubMed PMID: 30014812; PubMed Central PMCID: PMCPMC6169179.

31. Jones C, Ngasala B, Derua YA, Tarimo D, Reimer L, Bockarie M, et al. Lymphatic filariasis transmission in Rufiji District, southeastern Tanzania: infection status of the human population and mosquito vectors after twelve rounds of mass drug administration. Parasites & Vectors. 2018;11(1):588. doi: 10.1186/s13071-018-3156-2.

32. Budge PJ, Dorkenoo AM, Sodahlon YK, Fasuyi OB, Mathieu E. Ongoing surveillance for lymphatic filariasis in Togo: assessment of alternatives and nationwide reassessment of transmission status. Am J Trop Med Hyg. 2014;90(1):89-95. Epub 2013/11/06. doi: 10.4269/ajtmh.13-0407. PubMed PMID: 24189363; PubMed Central PMCID: PMCPMC3886434.

33. Dorkenoo MA, Bronzan R, Yehadji D, Tchalim M, Yakpa K, Etassoli S, et al. Surveillance for lymphatic filariasis after stopping mass drug administration in endemic districts of Togo, 2010-2015. Parasites & Vectors. 2018;11(1):244. Epub 2018/04/18. doi: 10.1186/s13071-018-2843-3. PubMed PMID: 29661231; PubMed Central PMCID: PMCPMC5902853.

34. Mathieu E, Dorkenoo A, Otogbe FK, Budge PJ, Sodahlon YK. A laboratory-based surveillance system for Wuchereria bancrofti in Togo: a practical model for resource-poor settings. Am J Trop Med Hyg. 2011;84(6):988-93. Epub 2011/06/03. doi: 10.4269/ajtmh.2011.10-0610. PubMed PMID: 21633038; PubMed Central PMCID: PMCPMC3110357.

35. Allen T, Taleo F, Graves PM, Wood P, Taleo G, Baker MC, et al. Impact of the Lymphatic Filariasis Control Program towards elimination of filariasis in Vanuatu, 1997–2006. Tropical Medicine and Health. 2017;45(1):8. doi: 10.1186/s41182-017-0047-8.

36. Schmaedick MA, Koppel AL, Pilotte N, Torres M, Williams SA, Dobson SL, et al. Molecular xenomonitoring using mosquitoes to map lymphatic filariasis after mass drug administration in American Samoa. PLoS Negl Trop Dis. 2014;8(8):e3087. doi: 10.1371/journal.pntd.0003087.

37. Irish SR, Al-Amin HM, Paulin HN, Mahmood ASMS, Khan RK, Muraduzzaman AKM, et al. Molecular xenomonitoring for Wuchereria bancrofti in Culex quinquefasciatus in two districts in Bangladesh supports transmission assessment survey findings. PLoS Negl Trop Dis. 2018;12(7):e0006574. doi: 10.1371/journal.pntd.0006574.

38. Abdel-Shafi IR, Shoeib EY, Attia SS, Rubio JM, Edmardash Y, El-Badry AA. Mosquito identification and molecular xenomonitoring of lymphatic filariasis in selected endemic areas in Giza and Qualioubiya Governorates, Egypt. J Egypt Soc Parasitol. 2016;46(1):93-100. Epub 2016/07/02. PubMed PMID: 27363044.

39. Moustafa MA, Salamah MMI, Thabet HS, Tawfik RA, Mehrez MM, Hamdy DM. Molecular xenomonitoring (MX) and transmission assessment survey (TAS) of lymphatic filariasis elimination in two villages, Menoufyia Governorate, Egypt. Eur J Clin Microbiol Infect Dis. 2017;36(7):1143-50. Epub 2017/02/06. doi: 10.1007/s10096-017-2901-3. PubMed PMID: 28155014.

40. Subramanian S, Jambulingam P, Chu BK, Sadanandane C, Vasuki V, Srividya A, et al. Application of a household-based molecular xenomonitoring strategy to evaluate the lymphatic filariasis elimination program in Tamil Nadu, India. PLoS Negl Trop Dis. 2017;11(4):e0005519. Epub 2017/04/14. doi: 10.1371/journal.pntd.0005519. PubMed PMID: 28406927; PubMed Central PMCID: PMCPMC5404881.

41. Beng TS, Ahmad R, Hisam RSR, Heng SK, Leaburi J, Ismail Z, et al. Molecular xenomonitoring of filarial infection in Malaysian mosquitoes under the national program for elimination of lymphatic filariasis. Southeast Asian Journal of Tropical Medicine and Public Health. 2016;47:617-24.

42. Reimer LJ, Thomsen EK, Tisch DJ, Henry-Halldin CN, Zimmerman PA, Baea ME, et al. Insecticidal Bed Nets and Filariasis Transmission in Papua New Guinea. New England Journal of Medicine. 2013;369(8):745-53. doi: 10.1056/NEJMoa1207594. PubMed PMID: 23964936.

43. Cho SH, Ma DW, Koo BR, Shin HE, Lee WK, Jeong BS, et al. Surveillance and vector control of lymphatic filariasis in the republic of Korea. Osong Public Health and Research Perspectives. 2012;3(3):145-50. doi: 10.1016/j.phrp.2012.07.008. PubMed PMID: 24159506.

44. Dorkenoo MA, de Souza DK, Apetogbo Y, Oboussoumi K, Yehadji D, Tchalim M, et al. Molecular xenomonitoring for post-validation surveillance of lymphatic filariasis in Togo: no evidence for active transmission. Parasites & Vectors. 2018;11(1):52. Epub 2018/01/25. doi: 10.1186/s13071-017-2611-9. PubMed PMID: 29361964; PubMed Central PMCID: PMCPMC5781303.
