# Supplementary material for: A systematic review of alternative surveillance approaches for lymphatic filariasis in low prevalence settings: Implications for post-validation settings
Source: PLoS Negl Trop Dis. 2020 May 12;14(5):e0008289. doi: 10.1371/journal.pntd.0008289 (PMC7217451; doi:10.1371/journal.pntd.0008289)
Supplement: S2 Table — (DOCX) [file pntd.0008289.s004.docx]

| **S2 Table. Human surveillance study results** | | | | | | | |
| --- | --- | --- | --- | --- | --- | --- | --- |
| **Reference** | **Country** | **Study date** | **Context** | **Study design** | **Age criteria** | **Sample size** | **Prevalence of infection (95% C.I. if stated)** |
| Mladonicky et al. 2009 [1] | American Samoa | 2006 | Post-MDA | Cross-sectional community survey | ≥5 years | 579 | BinaxNOW = 3.7-4.6%  MF = 0-0.6% (n.s.)  Bm14 Ab = 12.5-14.9% |
| Coutts et al. 2017 [2] | American Samoa | 2007 | Post-MDA | Cross-sectional community survey | ≥2 years | 1,881 | BinaxNow = 3.32% (2.44-4.51%) |
| Lau et al. 2014 [3] | American Samoa | 2010 | Post-MDA | Cross-sectional community survey | ≥18 years | 807 | Og4cC3 Ag>128 units = 0.75% (0.3-1.6%) Og4cC3 Ag>32 units = 3.2% (0.6-4.7%) Wb123 Ab = 8.1% (6.3-10.2%) Bm14 Ab = 17.9% (15.3-20.7%) |
| Lau et al. 2017 [4] | American Samoa | 2014 | Post-MDA | Cross-sectional occupational survey | ≥15 years | 602 | BinaxNOW/Og4C3 Ab = 1.2% (0.6-2.6%)  Bm14 Ab = 9.6% (7.5-12.3%)  Wb123 Ab = 10.5% (7.6-14.3%) |
|  |  |  |  | Cross-sectional community survey | ≥2 years | 476 | Fagali'I (n=58) BinaxNOW/Og4C3 Ab = 26.9% (17.3-39.4%)  Bm14 Ab = 43.4% (32.4-55.0%)  Wb123 Ab = 55.2% (39.6-69.8%) |
|  |  |  |  |  |  |  | Ili'ili/Vaitogi/Futiga (n=418) BinaxNOW/Og4C3 Ab = 4.0% (1.8-8.8%)  Bm14 Ab = 12.5% (7.1-21.1%)  Wb123 Ab = 18.5% (11.4-28.5%) |
|  |  |  |  | Cross-sectional school survey | 7-13 years | 283 | BinaxNOW 1.1% (0.2-3.1%) |
| Won et al. 2018 [5] | American Samoa | 2015 | Post-MDA | Longitudinal school survey | 5-10 years | 1,998 | TAS 1 BinaxNOW = 0.2% (n.s.)  Wb123 Ab = 1.0% (n.s.)  Bm14 Ab = 6.8% (n.s.) Bm33 Ab = 12.0% (n.s.) |
|  |  |  |  |  |  |  | TAS2 BinaxNOW = 0.1% (n.s.)  Wb123 Ab = 3.6% (but different testing platform used)  Bm14 Ab = 3.0% (n.s.)  Bm33 Ab = 7.8% (n.s.) |
| Sheel et al. 2018 [6] | American Samoa | 2016 | Post-MDA | Cross-sectional community survey | ≥8 years | 2,507 | Community survey (n=2,507) FTS = 6.2% (4.5-8.6%)  MF = 22/86 +ve |
|  |  |  |  |  |  |  | Standard school-based TAS (n=1,143) FTS = 0.7% (0.3-1.8%)  MF = 1/9 +ve |
| Huang et al. 2016 [7] | China | 1990 | Post-validation | Cross-sectional community survey | Children and adults | 430 | People born before eradication (n=200) IgG4 ELISA = 9.5% (n.s.) |
|  |  |  |  |  |  |  | Children born after eradication (n=230) IgG4 ELISA = 0.87% (n.s.) |
|  |  | 1995 | Post-validation | Cross-sectional community survey | Not stated | 2,001 | People from previously highly endemic area (n=760) IgG4 ELISA = 0.97% (n.s.) |
|  |  |  |  |  |  |  | People from previously lowly endemic area (n=1095) IgG4 ELISA = 0.27% (n.s.) |
|  |  |  |  |  |  |  | Microfilaraemia cases turning negative (n=69) IgG4 ELISA = 1.45% (n.s.) |
|  |  |  |  |  |  |  | Late stage filariasis cases (n=77) IgG4 ELISA = 0% (n.s.) |
|  |  | 1997 | Post-validation | Cross-sectional community survey | Not stated | 351 | Former high endemic area MF prevalence = 18.73% (n.s.)  IgG4 ELISA = 26.37% (n.s.) |
|  |  |  |  |  |  |  | Former moderate endemic area MF prevalence = 0.14% (n.s.)  IgG4 ELISA = 0.77% (n.s.) |
|  |  | 2002 | Post-validation | Cross-sectional school survey | Children | 542 | IgG4 ELISA = 0% (n.s.)  MF = 0% (n.s.) |
|  |  | 2002 and 2004 | Post-validation | Cross-sectional community survey | Children and adults | 762 | Microfilaraemia cases turning negative (n=419) IgG4 ELISA = 1.4% (n.s.)  MF = 0% (n.s.) |
|  |  |  |  |  |  |  | Children born after basic eradication of filariasis (n=143) IgG4 ELISA = 0% (n.s.) |
|  |  |  |  |  |  |  | Children from non-endemic regions (n=200) IgG4 ELISA = 0% (n.s.) |
|  |  | 2002-2008 | Post-validation | Longitudinal community survey | Not stated | 218 | 2002 (n=77) IgG4 ELISA = 9.09% (n.s.) |
|  |  |  |  |  |  |  | 2004 (n=78) IgG4 ELISA = 10.26% (n.s.) |
|  |  |  |  |  |  |  | 2008 (n=63) IgG4 ELISA = 1.58% (n.s.) |
|  |  | 2003 | Post-validation | Cross-sectional community survey | Not stated | 436 | Children born after basic eradication of filariasis (n=312) IgG4 ELISA = 0% (n.s.) |
|  |  |  |  |  |  |  | Late stage filariasis cases (n=48) IgG4 ELISA = 0% (n.s.) |
|  |  |  |  |  |  |  | Microfilaraemia cases turning negative (n=76) IgG4 ELISA = 0% (n.s.) |
|  |  | 2004 | Post-validation | Cross-sectional community survey | Not stated | 5,787 | People from previously low endemic area (n=2,744) IgG4 ELISA = 0% (n.s.) |
|  |  |  |  |  |  |  | People from previously moderate endemic area (n=800) IgG4 ELISA = 0% (n.s.) |
|  |  |  |  |  |  |  | People from previously high endemic area (n=2243) IgG4 ELISA = 0% (n.s.) |
| Huang et al. (2016) | China | Not stated | Post-validation | Cross-sectional community survey | Children and adults | 143 | People from previously highly endemic area (n=48) IgG4 ELISA = 12.5% (n.s.) |
|  |  |  |  |  |  |  | Microfilaraemia cases turning negative (n=61) IgG4 ELISA = 18.03% (n.s.) |
|  |  |  |  |  |  |  | Children born after basic eradication of filariasis (n=34) IgG4 ELISA = 17.65% (n.s.) |
| Itoh et al. 2007 [8] | China | 2004 | Post-validation | Cross-sectional school survey | 6 to 10 years (Yongjia) 5-15 years (Gaoan) | 10,409 | Yongjia  Urine IgG4 ELISA = 0.08% (n.s.) |
|  |  |  |  |  |  |  | Gaoan  Urine IgG4 ELISA = 0.35% (n.s.) |
| Moustafa et al. 2014 [9] | Egypt | 2012 | Post-MDA | Cross-sectional school survey | 6-7 years | 1,321 | BinaxNOW = 0% (n.s.)  Bm14 Ab = 2.2% (n.s.) |
|  |  |  |  | Cross-sectional community survey | 16-60 years | 75 | BinaxNOW = 0% (n.s.) |
| Ramzy et al. 2006 [10] | Egypt | Study year not reported | Post-MDA | Longitudinal community survey | ≥4 years | 1,808 | Giza BinaxNOW - not reported  MF = 0.3% (n.s.) |
|  |  |  |  |  |  |  | Qalubyia BinaxNOW = not reported  MF = 0% (n.s.) |
|  |  |  |  | Longitudinal school survey | 7 and 11 years | 1,653 | Giza BinaxNOW (Grade 1) = 0.4% (n.s.)  Bm14 (Grade 1) = 0.2% (0.0-0.5%)  Bm14 Ab = 1.4 (0.3-2.6%) |
|  |  |  |  |  |  |  | Qalubyia Grade 1 = 0% (n.s.)  Bm14 (Grade 1) = 0%  Bm14 Ab = 0% (n.s.) |
| Gass et al. 2011 [11] | French Polynesia | 2011 | Post-MDA | Cross-sectional community and school survey | 3-80 years | 1,383 | Bm14 Ab = 46.0%  PanLF = 14.0%  Urine SXP = 22.5%  BinaxNOW = 9.0%  Og4C3 Ag = 6.4%  MF = 3.8%  PCR = 2.2% |
| Won et al. 2018 [5] | Gambia | 2015 | Post-elimination | Cross-sectional community survey | ≥1 year | 2,612 | Wb123 Ab = 1.5% (1.1-2.1%)  Bm14 Ab = 1.5% (0.7-2.8%) |
| Gass et al. 2011 [11] | Ghana | 2011 | Post-MDA | Cross-sectional community and school survey | 3-80 years | 1,466 | Bm14 Ab = 9.9%  BinaxNOW = 6.7%  Og4C3 Ag = 8.9%  MF = 2.1%  PCR = 0.8% |
| Owusu et al. 2015 [12] | Ghana | 2008 | Post-MDA | Cross-sectional school survey | 6-7 and 10-11 years | 308 | ICT = 1.6% (n.s.)  Og4C3 Ag = 1.0% (n.s.)  Bm14 Ab = 4.9% (n.s.)  Blood PCR = 0% (n.s.) |
|  |  |  |  | Cross-sectional community survey | 3-80 years | 653 | ICT = 7.8% (n.s.)  MF = 1.7% (n.s.)  Og4C3 Ag = 12.2% (n.s.)  Bm14 Ab = 12.9% (n.s.)  Blood PCR = 0.9% (n.s.) |
| Gass et al. 2011 [11] | Haiti | 2011 | Post-MDA | Cross-sectional community and school survey | 3-80 years | 1,322 | Bm14 Ab = 53.1%  PanLF = 41.5%  Urine SXP = 18.5%  BinaxNOW = 21.2%  Og4C3 Ag = 18.8%  MF = 4.3%  PCR = 4.0% |
| Mehta et al. 2018 [13] | India | Study year not reported | Post-MDA | Cross-sectional community survey | ≥5 years | 290 | MF = 0.69% (n.s.)  BinaxNOW = 2.35% (n.s.) |
| Ramaiah et al. 2013 [14] | India | 2005-2008 | Post-MDA | Longitudinal community survey | Adults and children | 700 | MF = 0.9% (0.2-1.6%) at end of year one after stopping MDA. Trend remained stable in subsequent three years ICT = 1.2% (0.0-5.0%) at four years after stopping MDA. |
| Swaminathan et al. 2017 [15] | India | 2015-2017 | Post-MDA | Cross-sectional community survey | ≥2 years | 35,582 | MF = 0.2% (n.s.)  Og4C3 Ag = 2.3% (n.s.) |
| Garchitorena et al. 2018 [16] | Madagascar | 2016 | Post-MDA | Cross-sectional community survey | ≥5 years | 545 | FTS = 15.78% (12.88-19.18%) in community survey. Ranging from 2.38% in 8-14 years to 27.78% in 46-90 years.  Compared to 0.81% (0.3-1.99%) in sentinel and spot-check survey. |
| Coulibaly et al. 2015 [17] | Mali | 2007 | Post-MDA | Longitudinal community survey | ≥2 years | 760 | ICT = 0% (n.s.)  MF = 0% (n.s.) – children only |
| Coulibaly et al. 2016 [18] | Mali | 2009-2013 | Post-MDA | Longitudinal community survey | 6-7 years | 3,457 | 2009  BinaxNOW = 0% (0.00-1.64%)  MF = 0% (n.s.)  Wb PCR = 0 % (n.s.) |
|  |  |  |  |  |  |  | 2011  BinaxNOW = 2.7% (1.24-5.37%)  MF = 0% (0.00-40.23%) |
|  |  |  |  |  |  |  | 2012  BinaxNOW = 3.9% (2.04-7.00%)  MF = 0% (0.00-32.15%)  Wb123Ab = 1.8% (0.65-4.27%)  Og4C3 Ag = 1.8% (0.65-4.27%) |
|  |  |  |  |  |  |  | 2013  BinaxNOW = 4.5% (2.60-7.66%)  MF = 0% (0.00-26.76%) |
| Coulibaly et al. 2016 [18] | Mali | 2009-2013 | Post-MDA | Longitudinal community survey | ≥8 years | 1,184 | 2009 BinaxNOW = 4.9% (3.53-6.67%)  MF = 2.6% (0.06-13.48%)  Wb PCR = 5.13% (0.89-18.63%) |
|  |  |  |  |  |  |  | 2011 BinaxNOW = 3.5% (2.40-5.12%)  MF = 10.7% (2.81-29.37%) |
|  |  |  |  |  |  |  | 2012 BinaxNOW = 2.8% (2.08-3.65%)  MF = 0% (0.00-8.89%)  Wb123Ab = 4.7% (0.81-17.06%)  Og4C3 Ag = 4% (0.70-14.86%) |
| Richards et al. 2011 [19] | Nigeria | 2009 | Post-MDA | Longitudinal community survey | ≥2 years | 1,720 | ICT = 7.4% (n.s.)  MF = 0.9% (n.s.) |
| Mitja et al. 2018 [20] | Papua New Guinea | 2015 | Post-MDA | Cross sectional community survey | 10-79 years | 854 | BinaxNOW = 1.1% (0.6-2.0%) |
| Tisch et al. 2008 [21] | Papua New Guinea | 1999 | Post-MDA | Longitudinal community survey | All ages | 189 | MF= 4% (n.s.)  Og4C3Ag = 78% (n.s.)  Bm14 Ab = 49% (n.s.) |
| Joseph et al. 2011 [22] | Samoa | 2007 | Post-MDA | Cross-sectional community survey | Any age | 6,648 | MF = Up to 2%  BinaxNOW = Up to 7.3%  Bm14 Ab = 30.7% (n.s.) |
| Joseph et al. 2011 [23] | Samoa | 2008 | Post-MDA | Cross-sectional community survey | ≥2 years | 2,474 | BinaxNOW = 1.6 - 14.6%  MF = 0 - 3.2%  Bm14 Ab = 34.3 - 74.9% |
| Harrington et al. 2013 [24] | Solomon Islands | 2011 | Post-validation | Cross-sectional community survey | Adults and children | 307 | Og4C3 Ag = 0.3% (n.s.)  MF = 0% (n.s.) |
| Chandrasena et al. 2016 [25] | Sri Lanka | 2009-2015 | Post-MDA | Longitudinal community survey | 4-80 years | 2,461 | 2009/10 MF = 0.3% (n.s.) |
|  |  |  |  |  |  |  | 2013-15 MF = 0.1% (n.s.) |
| Gass et al. 2011 [11] | Sri Lanka | 2011 | Post-MDA | Cross-sectional community and school survey | 3-80 years | 1,477 | PanLF = 7.2%  BinaxNOW = 3.0%  Og4C3 Ag = 0.5%  MF = 0.4%  PCR =0.2% |
| Rao et al. 2014 [26] | Sri Lanka | 2011-2013 | Post-MDA | Cross-sectional community survey | ≥10 years | 7,156 | MF = 0-0.9%  BinaxNOW = 0-3.4%  Subset of PHIs reported Bm14Ab – see main paper |
| Rao et al. 2016 [27] | Sri Lanka | 2013 | Post-MDA | Cross-sectional community survey | 2-70 years | 12,977 | MF = 0% (0.02-0.09%) |
| Chandrasena et al. 2016 [25] | Sri Lanka | 2015 | Post-MDA | Cross-sectional community survey | 7-12 years | 250 | Brugia Rapid = 1.6% (n.s.) |
| Rao et al. 2017 [28] | Sri Lanka | 2015-2017 | Post-MDA | Cross-sectional community survey | ≥10 years | 3,123 | BinaxNOW = 0-1.5%  MF = 0-0.2% (n.s.) |
|  |  |  |  | Cross-sectional school survey | 6-8 years | 2,227 | BinaxNOW = 0.-1.1%  MF = 0-0.3%  Bm14 Ab = 0-4.2% |
| Rahman et al. 2019 [29] | Sri Lanka | Study year not reported | Post-TAS | Cross-sectional school survey | 5-13 years | 2,301 | Urine IgG4 = 1.78% (1.31-2.41%) |
|  |  |  |  | Cross-sectional community survey | 5-84 years | 630 | MF = 0% (n.s.)  FTS = 1.43% (0.75-2.70%) |
| Rao et al. 2014 [26] | Sri Lanka | Study year not reported | Post-MDA | Cross-sectional school survey | Grade 1 and 2 | 17,000 | BinaxNOW = 0-0.8%  BM14 Ab = 0-6.9% |
| Rao et al. 2018 [30] | Sri Lanka | 2015 | Post-MDA | Cross-sectional community survey | ≥2 years | 16,927 | MF = 0.6% (0.47-0.71%) |
|  |  |  |  | Cross-sectional school survey | First and second grade children | 401 | BinaxNOW = 1.2% (0.5-2.8%)  MF = 0.2% (0.0-1.4%)  Bm14 Ab = 5.7% (3.7-8.4%) |
|  |  |  |  | Cross-sectional community survey | 10-70 years | 528 | BinaxNOW = 3.0% (1.8-4.9%)  MF = 1.1% (0.5-2.5%) |
| Gass et al. 2011 [11] | Tanzania | 2011 | Post-MDA | Cross-sectional community and school survey | 3-80 years | 1,384 | Urine SXP = 20.9%  BinaxNOW = 8.1%  Og4C3 Ag = 8.2%  PCR = 0.8% |
| Budge et al. 2014 [31] | Togo | 2006-2007 | Post-MDA | Longitudinal laboratory surveillance study | Adults | 6,509 | MF = 0.02% (n.s.) |
|  |  | 2010 | Post-MDA | Cross-sectional community survey | Adults | 7,800 | BinaxNOW = 0.3% (n.s.) |
|  |  | 2010-2011 | Post-MDA | Longitudinal health facility surveillance study | Adults | 2,880 | Og4C3Ag ELISA = 0% (n.s.) |
| Mathieu et al. 2011 [32] | Togo | 2006-2007 | Post-MDA | Longitudinal laboratory surveillance study | Age criteria not stated | 8,050 | MF = 0.02% (n.s.) |
| Dorkenoo et al. 2018 [33] | Togo | 2010-2015 | Post-MDA | Cross-sectional active surveillance of positive cases | Children and adults | 40 | 4 cases of MF identified. 1 further MF case identified among 287 contacts screened. Of these 5 cases, 4 were considered to be imported from another country. |
| Joseph et al. 2011 [22] | Tonga | 2007 | Post-MDA | Cross-sectional school survey | 5-6 years | 797 | BinaxNOW = 0% (n.s.)  Bm14 Ab = 6.3% (n.s.) |
| Gass et al. 2011[11] | Tuvalu | 2011 | Post-MDA | Cross-sectional community and school survey | 3-80 years | 1,481 | PanLF = 25.2%  Urine SXP = 20.1%  BinaxNOW = 5.0%  Og4C3 Ag = 4.9%  MF = 0.1%  PCR = 0.3% |
| Allen at al. 2017 [34] | Vanuatu | 2005-2006 | Post-MDA | Cross-sectional community survey | ≥1 year | 7,657 | BinaxNOW = 0.16% (n.s.) |
| Joseph et al. 2011 [22] | Vanuatu | 2007 | Post-MDA | Cross-sectional school survey | 5-6 years | 3,840 | BinaxNOW = 0% (n.s.)  Bm14 Ab = 6.0% (n.s.) |

**References**

1. Mladonicky JM, King JD, Liang JL, Chambers E, Pa'au M, Schmaedick MA, et al. Assessing transmission of lymphatic filariasis using parasitologic, serologic, and entomologic tools after mass drug administration in American Samoa. Am J Trop Med Hyg. 2009;80(5):769-73. Epub 2009/05/02. PubMed PMID: 19407122.

2. Coutts SP, King JD, Pa'au M, Fuimaono S, Roth J, King MR, et al. Prevalence and risk factors associated with lymphatic filariasis in American Samoa after mass drug administration. Tropical Medicine and Health. 2017;45:22. Epub 2017/08/11. doi: 10.1186/s41182-017-0063-8. PubMed PMID: 28794687; PubMed Central PMCID: PMCPMC5543440.

3. Lau CL, Won KY, Becker L, Soares Magalhaes RJ, Fuimaono S, Melrose W, et al. Seroprevalence and spatial epidemiology of lymphatic filariasis in American Samoa after successful mass drug administration. PLoS Negl Trop Dis. 2014;8(11):e3297-e. doi: 10.1371/journal.pntd.0003297. PubMed PMID: 25393716.

4. Lau CL, Sheridan S, Ryan S, Roineau M, Andreosso A, Fuimaono S, et al. Detecting and confirming residual hotspots of lymphatic filariasis transmission in American Samoa 8 years after stopping mass drug administration. PLoS Negl Trop Dis. 2017;11(9):e0005914. doi: 10.1371/journal.pntd.0005914.

5. Won KY, Robinson K, Hamlin KL, Tufa J, Seespesara M, Wiegand RE, et al. Comparison of antigen and antibody responses in repeat lymphatic filariasis transmission assessment surveys in American Samoa. PLoS Negl Trop Dis. 2018;12(3):e0006347. doi: 10.1371/journal.pntd.0006347.

6. Sheel M, Sheridan S, Gass K, Won K, Fuimaono S, Kirk M, et al. Identifying residual transmission of lymphatic filariasis after mass drug administration: Comparing school-based versus community-based surveillance - American Samoa, 2016. PLoS Negl Trop Dis. 2018;12(7):e0006583. doi: 10.1371/journal.pntd.0006583.

7. Huang BC, Li J, Hu YX, Duan JH, Yin K, Xiao T, et al. Study on application of filarial specific IgG4 kit in disease surveillance of lymphatic filariasis. Int J Clin Exp Med. 2016;9:4332-9.

8. Itoh M, Wu W, Sun D, Yao L, Li Z, Islam MZ, et al. Confirmation of elimination of lymphatic filariasis by an IgG4 enzyme-linked immunosorbent assay with urine samples in Yongjia, Zhejiang Province and Gaoan, Jiangxi Province, People's Republic of China. Am J Trop Med Hyg. 2007;77(2):330-3. Epub 2007/08/11. PubMed PMID: 17690407.

9. Moustafa MA, Thabet HS, Saad GA, El-Setouhy M, Mehrez M, Hamdy DM. Surveillance of lymphatic filariasis 5 years after stopping mass drug administration in Menoufiya Governorate, Egypt. East Mediterr Health J. 2014;20(5):295-9. Epub 2014/06/22. PubMed PMID: 24952286.

10. Ramzy RM, El Setouhy M, Helmy H, Ahmed ES, Abd Elaziz KM, Farid HA, et al. Effect of yearly mass drug administration with diethylcarbamazine and albendazole on bancroftian filariasis in Egypt: a comprehensive assessment. The Lancet. 2006;367(9515):992-9. Epub 2006/03/28. doi: 10.1016/s0140-6736(06)68426-2. PubMed PMID: 16564361.

11. Gass K, Beau de Rochars MVE, Boakye D, Bradley M, Fischer PU, Gyapong J, et al. A Multicenter Evaluation of Diagnostic Tools to Define Endpoints for Programs to Eliminate Bancroftian Filariasis. PLOS Neglected Tropical Diseases. 2012;6(1):e1479. doi: 10.1371/journal.pntd.0001479.

12. Owusu IO, de Souza DK, Anto F, Wilson MD, Boakye DA, Bockarie MJ, et al. Evaluation of human and mosquito based diagnostic tools for defining endpoints for elimination of Anopheles transmitted lymphatic filariasis in Ghana. Trans R Soc Trop Med Hyg. 2015;109(10):628-35. Epub 2015/09/20. doi: 10.1093/trstmh/trv070. PubMed PMID: 26385935.

13. Mehta PK, Rauniyar R, Gupta BP. Microfilaria persistent foci during post MDA and the risk assessment of resurgence in India. Tropical Medicine and Health. 2018;46:25-. doi: 10.1186/s41182-018-0107-8. PubMed PMID: 30026669.

14. Ramaiah KD, Vanamail P. Surveillance of lymphatic filariasis after stopping ten years of mass drug administration in rural communities in south India. Trans R Soc Trop Med Hyg. 2013;107(5):293-300. Epub 2013/02/28. doi: 10.1093/trstmh/trt011. PubMed PMID: 23442572.

15. Swaminathan S, Perumal V, Adinarayanan S, Kaliannagounder K, Rengachari R, Purushothaman J. Epidemiological assessment of eight rounds of mass drug administration for lymphatic filariasis in India: Implications for monitoring and evaluation. PLoS Negl Trop Dis. 2012;6(11):e1926. doi: 10.1371/journal.pntd.0001926.

16. Garchitorena A, Raza-Fanomezanjanahary EM, Mioramalala SA, Chesnais CB, Ratsimbasoa CA, Ramarosata H, et al. Towards elimination of lymphatic filariasis in southeastern Madagascar: Successes and challenges for interrupting transmission. PLoS Negl Trop Dis. 2018;12(9):e0006780. doi: 10.1371/journal.pntd.0006780.

17. Coulibaly YI, Dembele B, Diallo AA, Konate S, Dolo H, Coulibaly SY, et al. The Impact of Six Annual Rounds of Mass Drug Administration on Wuchereria bancrofti Infections in Humans and in Mosquitoes in Mali. Am J Trop Med Hyg. 2015;93(2):356-60. Epub 2015/06/03. doi: 10.4269/ajtmh.14-0516. PubMed PMID: 26033027; PubMed Central PMCID: PMCPMC4530761.

18. Coulibaly YI, Coulibaly SY, Dolo H, Konate S, Diallo AA, Doumbia SS, et al. Dynamics of antigenemia and transmission intensity of Wuchereria bancrofti following cessation of mass drug administration in a formerly highly endemic region of Mali. Parasites & Vectors. 2016;9(1):628-. doi: 10.1186/s13071-016-1911-9. PubMed PMID: 27912789.

19. Richards FO, Eigege A, Miri ES, Kal A, Umaru J, Pam D, et al. Epidemiological and entomological evaluations after six years or more of mass drug administration for lymphatic filariasis elimination in Nigeria. PLoS Negl Trop Dis. 2011;5(10):e1346. Epub 2011/10/25. doi: 10.1371/journal.pntd.0001346. PubMed PMID: 22022627; PubMed Central PMCID: PMCPMC3191131.

20. Mitjà O, Paru R, Hays R, Griffin L, Laban N, Samson M, et al. The Impact of a Filariasis Control Program on Lihir Island, Papua New Guinea. PLOS Neglected Tropical Diseases. 2011;5(8):e1286. doi: 10.1371/journal.pntd.0001286.

21. Tisch DJ, Bockarie MJ, Dimber Z, Kiniboro B, Tarongka N, Hazlett FE, et al. Mass drug administration trial to eliminate lymphatic filariasis in Papua New Guinea: changes in microfilaremia, filarial antigen, and Bm14 antibody after cessation. Am J Trop Med Hyg. 2008;78(2):289-93. Epub 2008/02/08. PubMed PMID: 18256431; PubMed Central PMCID: PMCPMC2590750.

22. Joseph H, Maiava F, Naseri T, Taleo F, ake M, Capuano C, et al. Application of the Filariasis CELISA Antifilarial IgG(4) Antibody Assay in surveillance in lymphatic filariasis elimination programmes in the South Pacific. J Trop Med. 2011;2011:492023. Epub 2011/10/01. doi: 10.1155/2011/492023. PubMed PMID: 21961018; PubMed Central PMCID: PMCPMC3180782.

23. Joseph H, Maiava F, Naseri T, Silva U, Lammie P, Melrose W. Epidemiological assessment of continuing transmission of lymphatic filariasis in Samoa. Ann Trop Med Parasitol. 2011;105(8):567-78. Epub 2012/02/14. doi: 10.1179/2047773211y.0000000008. PubMed PMID: 22325816; PubMed Central PMCID: PMCPMC4089807.

24. Harrington H, Asugeni J, Jimuru C, Gwalaa J, Ribeyro E, Bradbury R, et al. A practical strategy for responding to a case of lymphatic filariasis post-elimination in Pacific Islands. Parasites & Vectors. 2013;6(1):218. doi: 10.1186/1756-3305-6-218.

25. Chandrasena NT, Premaratna R, Samarasekera DS, de Silva NR. Surveillance for transmission of lymphatic filariasis in Colombo and Gampaha districts of Sri Lanka following mass drug administration. Trans R Soc Trop Med Hyg. 2016;110(10):620-2. Epub 2016/11/07. doi: 10.1093/trstmh/trw067. PubMed PMID: 27816936.

26. Rao RU, Nagodavithana KC, Samarasekera SD, Wijegunawardana AD, Premakumara WD, Perera SN, et al. A comprehensive assessment of lymphatic filariasis in Sri Lanka six years after cessation of mass drug administration. PLoS Negl Trop Dis. 2014;8(11):e3281. Epub 2014/11/14. doi: 10.1371/journal.pntd.0003281. PubMed PMID: 25393404; PubMed Central PMCID: PMCPMC4230885.

27. Rao RU, Samarasekera SD, Nagodavithana KC, Punchihewa MW, Dassanayaka TD, P KDG, et al. Programmatic use of molecular xenomonitoring at the level of evaluation units to assess persistence of lymphatic filariasis in Sri Lanka. PLoS Negl Trop Dis. 2016;10(5):e0004722. Epub 2016/05/20. doi: 10.1371/journal.pntd.0004722. PubMed PMID: 27196431; PubMed Central PMCID: PMCPMC4873130.

28. Rao RU, Samarasekera SD, Nagodavithana KC, Dassanayaka TDM, Punchihewa MW, Ranasinghe USB, et al. Reassessment of areas with persistent lymphatic filariasis nine years after cessation of mass drug administration in Sri Lanka. PLoS Negl Trop Dis. 2017;11(10):e0006066. Epub 2017/10/31. doi: 10.1371/journal.pntd.0006066. PubMed PMID: 29084213; PubMed Central PMCID: PMCPMC5679644.

29. Rahman MA, Yahathugoda TC, Tojo B, Premaratne P, Nagaoka F, Takagi H, et al. A surveillance system for lymphatic filariasis after its elimination in Sri Lanka. Parasitol Int. 2019;68(1):73-8. Epub 2018/10/12. doi: 10.1016/j.parint.2018.10.003. PubMed PMID: 30308253.

30. Rao RU, Samarasekera SD, Nagodavithana KC, Goss CW, Punchihewa MW, Dassanayaka TDM, et al. Comprehensive Assessment of a Hotspot with Persistent Bancroftian Filariasis in Coastal Sri Lanka. Am J Trop Med Hyg. 2018;99(3):735-42. Epub 2018/07/18. doi: 10.4269/ajtmh.18-0169. PubMed PMID: 30014812; PubMed Central PMCID: PMCPMC6169179.

31. Budge PJ, Dorkenoo AM, Sodahlon YK, Fasuyi OB, Mathieu E. Ongoing surveillance for lymphatic filariasis in Togo: assessment of alternatives and nationwide reassessment of transmission status. Am J Trop Med Hyg. 2014;90(1):89-95. Epub 2013/11/06. doi: 10.4269/ajtmh.13-0407. PubMed PMID: 24189363; PubMed Central PMCID: PMCPMC3886434.

32. Mathieu E, Dorkenoo A, Otogbe FK, Budge PJ, Sodahlon YK. A laboratory-based surveillance system for Wuchereria bancrofti in Togo: a practical model for resource-poor settings. Am J Trop Med Hyg. 2011;84(6):988-93. Epub 2011/06/03. doi: 10.4269/ajtmh.2011.10-0610. PubMed PMID: 21633038; PubMed Central PMCID: PMCPMC3110357.

33. Dorkenoo MA, Bronzan R, Yehadji D, Tchalim M, Yakpa K, Etassoli S, et al. Surveillance for lymphatic filariasis after stopping mass drug administration in endemic districts of Togo, 2010-2015. Parasites & Vectors. 2018;11(1):244. Epub 2018/04/18. doi: 10.1186/s13071-018-2843-3. PubMed PMID: 29661231; PubMed Central PMCID: PMCPMC5902853.

34. Allen T, Taleo F, Graves PM, Wood P, Taleo G, Baker MC, et al. Impact of the Lymphatic Filariasis Control Program towards elimination of filariasis in Vanuatu, 1997–2006. Tropical Medicine and Health. 2017;45(1):8. doi: 10.1186/s41182-017-0047-8.
