# Supplementary material for: A systematic review of alternative surveillance approaches for lymphatic filariasis in low prevalence settings: Implications for post-validation settings
Source: PLoS Negl Trop Dis. 2020 May 12;14(5):e0008289. doi: 10.1371/journal.pntd.0008289 (PMC7217451; doi:10.1371/journal.pntd.0008289)
Supplement: S3 Table — (DOCX) [file pntd.0008289.s005.docx]

| **S3 Table. Mosquito surveillance study results** | | | | | | |
| --- | --- | --- | --- | --- | --- | --- |
| **Reference** | **Country** | **Study date** | **Study design** | **Sampling method** | **Sample size** | **Results** |
| Schmaedick et al. 2014 [1] | American Samoa | 2011 | Cross-sectional survey | All major villages sampled on smaller islands. Random sample of villages on larger island. | 21,861 mosquitoes | Female Aedes polynesiensis = 0.28% (95% CI 0.20-0.39) Culex quinquefasciatus = 0.11% (95% CI 0.034-0.27) Aedes aegypti = 0.92% (95% CI 0.37-1.8) Aedes finlaya = 0.092% (95% CI 0.0028-0.48) Aedes upolensis = 0% (95% CI 0-0.073) |
| Irish et al. 2018 [2] | Bangladesh | 2016 | Cross-sectional survey | Random sample of 30 villages taken, followed by random sample of 6 HHs within each village. | 5,926 mosquitoes | Prevalence of W. bancrofti DNA = 0% |
| Ramzy et al. 2006 [3] | Egypt | Not stated | Longitudinal survey | Purposive sampling of 4 villages in 2 different regions with varying pre-MDA LF prevalence. Villages mapped and random sample of households taken. | 8,531 (total) mosquitoes | Giza  Mosquito infection rate 0.19% (0.08-0.38)  - Baseline 3.07% (2.38-3.88) |
|  |  |  |  |  |  | Qalubyia  Mosquito infection rate 0 (0-0.05)  - Baseline 4.37% (3.07-5.99) |
| Abdel-Shafi et al. 2016 [4] | Egypt | 2014-15 | Cross-sectional survey | Random sampling of houses in 3 villages in two endemic Governorates. | Not stated | Prevalence of W. bancrofti DNA = 0% |
| Moustafa et al. 2017 [5] | Egypt | 2014 | Cross-sectional survey | Purposive sampling of 2 villages identified as problematic areas following a failed TAS. | 7,970 mosquitoes | Prevalence of W. bancrofti DNA = 0% |
| Owusu et al. 2015 [6] | Ghana | 2008 | Cross-sectional survey | Purposive sampling of 2 communities followed by random household sampling. | 4,400 (total) mosquitoes | Anopheles: Maximum likelihood of infection = 4.33% (2.17-7.58)  Culex: Maximum likelihood of infection = 0.02% (0.008-0.130) |
| Ramaiah et al. 2013 [7] | India | 2005-2010 | Longitudinal survey | Purposive sampling of 5 villages in Tamil Nadu. Catch site sampling method not stated. | 10,842 (total) mosquitoes | Yr. 1: Mosquito infection rate 1.7% Yr. 2: Mosquito infection rate 0.8% Yr. 3: Mosquito infection rate 1.1% Yr. 4: Mosquito infection rate 1.9% Yr. 5: Mosquito infection rate 0.9% Yr. 6: Mosquito infection rate 0.5% |
| Subramanaian et al. 2017 [8] | India | 2012 | Longitudinal survey | Purposive sampling in two different hotspot areas followed by random sampling of households using a sampling interval proportional to the number of households in the EU. | 41,294 (total) mosquitoes | Hotspot EU 2010 Sample 1. W. bancrofti prevalence 2.7% (2.1-3.3) Sample 2. W. bancrofti prevalence 2.2% (1.7-2.8) Hotspot EU 2012 Sample 1. W. bancrofti prevalence 1.2% (0.8-1.6) Sample 2. W. bancrofti prevalence 0.6% (0.4-0.9) |
|  |  |  |  |  |  | PHC EU 2010 Sample 1. W. bancrofti prevalence 1.1% (0.8-1.5) Sample 2. W. bancrofti prevalence 0.9% (0.6-1.2) PHC EU 2012 Sample 1. W. bancrofti prevalence 0.3% (0.2-0.5) Sample 2. W. bancrofti prevalence 0.2% (0.1-0.4) |
| Mehta et al. 2018 [9] | India | Not stated | Cross-sectional survey | Purposive sampling at 6 houses in 4 streets of a village also undergoing human sampling. | 2,429 mosquitoes | Mosquito infection rate = 0.04% |
| Beng et al. 2016 [10] | Malaysia | Study date not stated | Cross-sectional survey | Purposive sampling from 21 endemic implementation units and 6 non-endemic implementation units. Catch site sampling method not stated. | 4,378 mosquitoes | None of the samples were infected with B. malayi or W. bancrofti. |
| Coulibaly et al. 2015 [11] | Mali | 2007 | Longitudinal survey | 6 villages selected, sampling method not stated. Only final result is post-MDA. | 4,680 mosquitoes | Infection frequency 0.02% |
| Coulibaly et al. 2016 [12] | Mali | 2009-2013 | Longitudinal survey | 6 villages selected, sampling method not stated. | 14,424 (total) mosquitoes | HLC 2009 Infection frequency 0.05% (0.01-0.18) HLC 2011 Infection frequency 0% HLC 2012 Infection frequency 0%  PSC 2012  Infection frequency 0% |
| Richards et al. 2011 [13] | Nigeria | 2009 | Longitudinal survey | Location sampling method not stated. 10 sentinel villages selected, followed by convenience sample of households willing to participate. | 4,398 mosquitoes | Infection frequency 0.4% |
| Reimer et al. 2013[14] | Papua New Guinea | 2007-2008 | Longitudinal survey | 5 villages selected, sampling method not stated. | 20,345 mosquitoes | Mosquito infectivity rate = 1.8% |
| Cho et al. 2012 [15] | South Korea | 2009 | Cross-sectional survey | 6 formerly endemic sites selected, sampling method not stated. | 5,380 mosquitoes | Mosquito infectivity rate = 0% |
| Rao et al. 2014 [16] | Sri Lanka | Study date not stated | Cross-sectional survey | Systematic sampling to sample all areas in each administrative sub-tier of included PHI areas. Followed by random sampling of location for catch sites. | 69,680 mosquitoes | Mosquito infectivity rate range 0% - 1.56%. Upper confidence limits for filarial DNA rates were ≥1% in 5 of 19 PHIs surveyed. |
| Rao et al. 2016 [17] | Sri Lanka | 2013-2014 | Cross-sectional survey | Random sampling of 30 PHMs selected as evaluation areas (EAs) followed by random sampling of households. | 28,717 mosquitoes | Filarial DNA rates = 0.36% (0.29-0.45%). |
| Rao et al. 2017 [18] | Sri Lanka | 2011-2016 | Longitudinal survey | Systematic sampling scheme in 6 sentinel PHI areas, followed by random sampling of households. | 48,301 mosquitoes | Mosquito infectivity rate range 0.26% - 1.50%. |
| Rao et al. 2018 [19] | Sri Lanka | 2015-2016 | Cross-sectional survey | Purposive sampling of a potential hotspot area followed by random sampling of catch sites. | 7,750 (total) mosquitoes | 2015: Filarial DNA prevalence 5.2% (4.2-6.3%). 2016: Filarial DNA prevalence 3.0% (2.3-3.8%). |
| Mitja et al. 2018  [20] | Tanzania | 2015 | Cross-sectional survey | Random sampling of villages in Rufiji district followed by purposive sampling of houses with thatched roofs and open eaves | 1,650 mosquitoes | Mosquito infectivity rate = 0% |
| Dorkenoo et al. 2018 [21] | Togo | 2015 | Cross-sectional | Random sampling of villages and purposive selection of villages with previous MF-positive cases, followed by random household sampling. | 10,872 mosquitoes | Mosquito infectivity rate = 0%. |

**References**

1. Schmaedick MA, Koppel AL, Pilotte N, Torres M, Williams SA, Dobson SL, et al. Molecular xenomonitoring using mosquitoes to map lymphatic filariasis after mass drug administration in American Samoa. PLoS Negl Trop Dis. 2014;8(8):e3087. doi: 10.1371/journal.pntd.0003087.

2. Irish SR, Al-Amin HM, Paulin HN, Mahmood ASMS, Khan RK, Muraduzzaman AKM, et al. Molecular xenomonitoring for Wuchereria bancrofti in Culex quinquefasciatus in two districts in Bangladesh supports transmission assessment survey findings. PLoS Negl Trop Dis. 2018;12(7):e0006574. doi: 10.1371/journal.pntd.0006574.

3. Ramzy RM, El Setouhy M, Helmy H, Ahmed ES, Abd Elaziz KM, Farid HA, et al. Effect of yearly mass drug administration with diethylcarbamazine and albendazole on bancroftian filariasis in Egypt: a comprehensive assessment. The Lancet. 2006;367(9515):992-9. Epub 2006/03/28. doi: 10.1016/s0140-6736(06)68426-2. PubMed PMID: 16564361.

4. Abdel-Shafi IR, Shoeib EY, Attia SS, Rubio JM, Edmardash Y, El-Badry AA. Mosquito identification and molecular xenomonitoring of lymphatic filariasis in selected endemic areas in Giza and Qualioubiya Governorates, Egypt. J Egypt Soc Parasitol. 2016;46(1):93-100. Epub 2016/07/02. PubMed PMID: 27363044.

5. Moustafa MA, Salamah MMI, Thabet HS, Tawfik RA, Mehrez MM, Hamdy DM. Molecular xenomonitoring (MX) and transmission assessment survey (TAS) of lymphatic filariasis elimination in two villages, Menoufyia Governorate, Egypt. Eur J Clin Microbiol Infect Dis. 2017;36(7):1143-50. Epub 2017/02/06. doi: 10.1007/s10096-017-2901-3. PubMed PMID: 28155014.

6. Owusu IO, de Souza DK, Anto F, Wilson MD, Boakye DA, Bockarie MJ, et al. Evaluation of human and mosquito based diagnostic tools for defining endpoints for elimination of Anopheles transmitted lymphatic filariasis in Ghana. Trans R Soc Trop Med Hyg. 2015;109(10):628-35. Epub 2015/09/20. doi: 10.1093/trstmh/trv070. PubMed PMID: 26385935.

7. Ramaiah KD, Vanamail P. Surveillance of lymphatic filariasis after stopping ten years of mass drug administration in rural communities in south India. Trans R Soc Trop Med Hyg. 2013;107(5):293-300. Epub 2013/02/28. doi: 10.1093/trstmh/trt011. PubMed PMID: 23442572.

8. Subramanian S, Jambulingam P, Chu BK, Sadanandane C, Vasuki V, Srividya A, et al. Application of a household-based molecular xenomonitoring strategy to evaluate the lymphatic filariasis elimination program in Tamil Nadu, India. PLoS Negl Trop Dis. 2017;11(4):e0005519. Epub 2017/04/14. doi: 10.1371/journal.pntd.0005519. PubMed PMID: 28406927; PubMed Central PMCID: PMCPMC5404881.

9. Mehta PK, Rauniyar R, Gupta BP. Microfilaria persistent foci during post MDA and the risk assessment of resurgence in India. Tropical Medicine and Health. 2018;46:25-. doi: 10.1186/s41182-018-0107-8. PubMed PMID: 30026669.

10. Beng TS, Ahmad R, Hisam RSR, Heng SK, Leaburi J, Ismail Z, et al. Molecular xenomonitoring of filarial infection in Malaysian mosquitoes under the national program for elimination of lymphatic filariasis. Southeast Asian Journal of Tropical Medicine and Public Health. 2016;47:617-24.

11. Coulibaly YI, Dembele B, Diallo AA, Konate S, Dolo H, Coulibaly SY, et al. The Impact of Six Annual Rounds of Mass Drug Administration on Wuchereria bancrofti Infections in Humans and in Mosquitoes in Mali. Am J Trop Med Hyg. 2015;93(2):356-60. Epub 2015/06/03. doi: 10.4269/ajtmh.14-0516. PubMed PMID: 26033027; PubMed Central PMCID: PMCPMC4530761.

12. Coulibaly YI, Coulibaly SY, Dolo H, Konate S, Diallo AA, Doumbia SS, et al. Dynamics of antigenemia and transmission intensity of Wuchereria bancrofti following cessation of mass drug administration in a formerly highly endemic region of Mali. Parasites & Vectors. 2016;9(1):628-. doi: 10.1186/s13071-016-1911-9. PubMed PMID: 27912789.

13. Richards FO, Eigege A, Miri ES, Kal A, Umaru J, Pam D, et al. Epidemiological and entomological evaluations after six years or more of mass drug administration for lymphatic filariasis elimination in Nigeria. PLoS Negl Trop Dis. 2011;5(10):e1346. Epub 2011/10/25. doi: 10.1371/journal.pntd.0001346. PubMed PMID: 22022627; PubMed Central PMCID: PMCPMC3191131.

14. Reimer LJ, Thomsen EK, Tisch DJ, Henry-Halldin CN, Zimmerman PA, Baea ME, et al. Insecticidal Bed Nets and Filariasis Transmission in Papua New Guinea. New England Journal of Medicine. 2013;369(8):745-53. doi: 10.1056/NEJMoa1207594. PubMed PMID: 23964936.

15. Cho SH, Ma DW, Koo BR, Shin HE, Lee WK, Jeong BS, et al. Surveillance and vector control of lymphatic filariasis in the republic of Korea. Osong Public Health and Research Perspectives. 2012;3(3):145-50. doi: 10.1016/j.phrp.2012.07.008. PubMed PMID: 24159506.

16. Rao RU, Nagodavithana KC, Samarasekera SD, Wijegunawardana AD, Premakumara WD, Perera SN, et al. A comprehensive assessment of lymphatic filariasis in Sri Lanka six years after cessation of mass drug administration. PLoS Negl Trop Dis. 2014;8(11):e3281. Epub 2014/11/14. doi: 10.1371/journal.pntd.0003281. PubMed PMID: 25393404; PubMed Central PMCID: PMCPMC4230885.

17. Rao RU, Samarasekera SD, Nagodavithana KC, Punchihewa MW, Dassanayaka TD, P KDG, et al. Programmatic use of molecular xenomonitoring at the level of evaluation units to assess persistence of lymphatic filariasis in Sri Lanka. PLoS Negl Trop Dis. 2016;10(5):e0004722. Epub 2016/05/20. doi: 10.1371/journal.pntd.0004722. PubMed PMID: 27196431; PubMed Central PMCID: PMCPMC4873130.

18. Rao RU, Samarasekera SD, Nagodavithana KC, Dassanayaka TDM, Punchihewa MW, Ranasinghe USB, et al. Reassessment of areas with persistent lymphatic filariasis nine years after cessation of mass drug administration in Sri Lanka. PLoS Negl Trop Dis. 2017;11(10):e0006066. Epub 2017/10/31. doi: 10.1371/journal.pntd.0006066. PubMed PMID: 29084213; PubMed Central PMCID: PMCPMC5679644.

19. Rao RU, Samarasekera SD, Nagodavithana KC, Goss CW, Punchihewa MW, Dassanayaka TDM, et al. Comprehensive Assessment of a Hotspot with Persistent Bancroftian Filariasis in Coastal Sri Lanka. Am J Trop Med Hyg. 2018;99(3):735-42. Epub 2018/07/18. doi: 10.4269/ajtmh.18-0169. PubMed PMID: 30014812; PubMed Central PMCID: PMCPMC6169179.

20. Mitjà O, Paru R, Hays R, Griffin L, Laban N, Samson M, et al. The Impact of a Filariasis Control Program on Lihir Island, Papua New Guinea. PLOS Neglected Tropical Diseases. 2011;5(8):e1286. doi: 10.1371/journal.pntd.0001286.

21. Dorkenoo MA, de Souza DK, Apetogbo Y, Oboussoumi K, Yehadji D, Tchalim M, et al. Molecular xenomonitoring for post-validation surveillance of lymphatic filariasis in Togo: no evidence for active transmission. Parasites & Vectors. 2018;11(1):52. Epub 2018/01/25. doi: 10.1186/s13071-017-2611-9. PubMed PMID: 29361964; PubMed Central PMCID: PMCPMC5781303.
